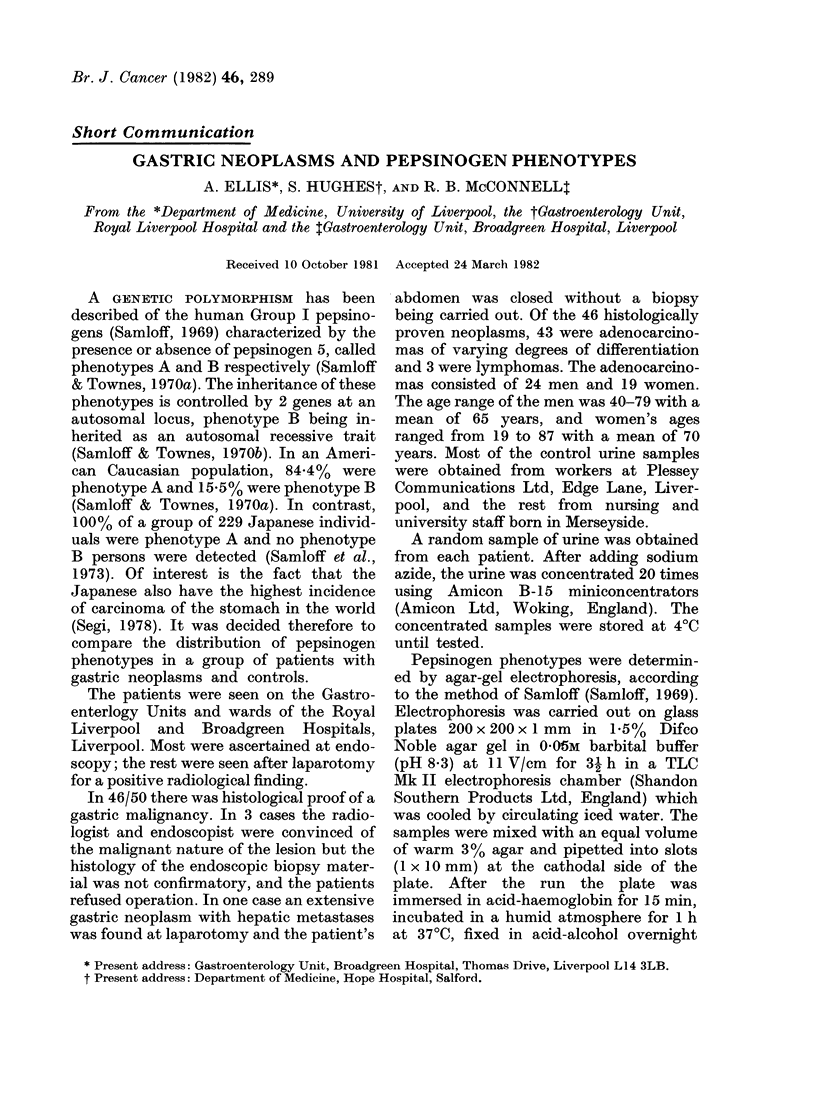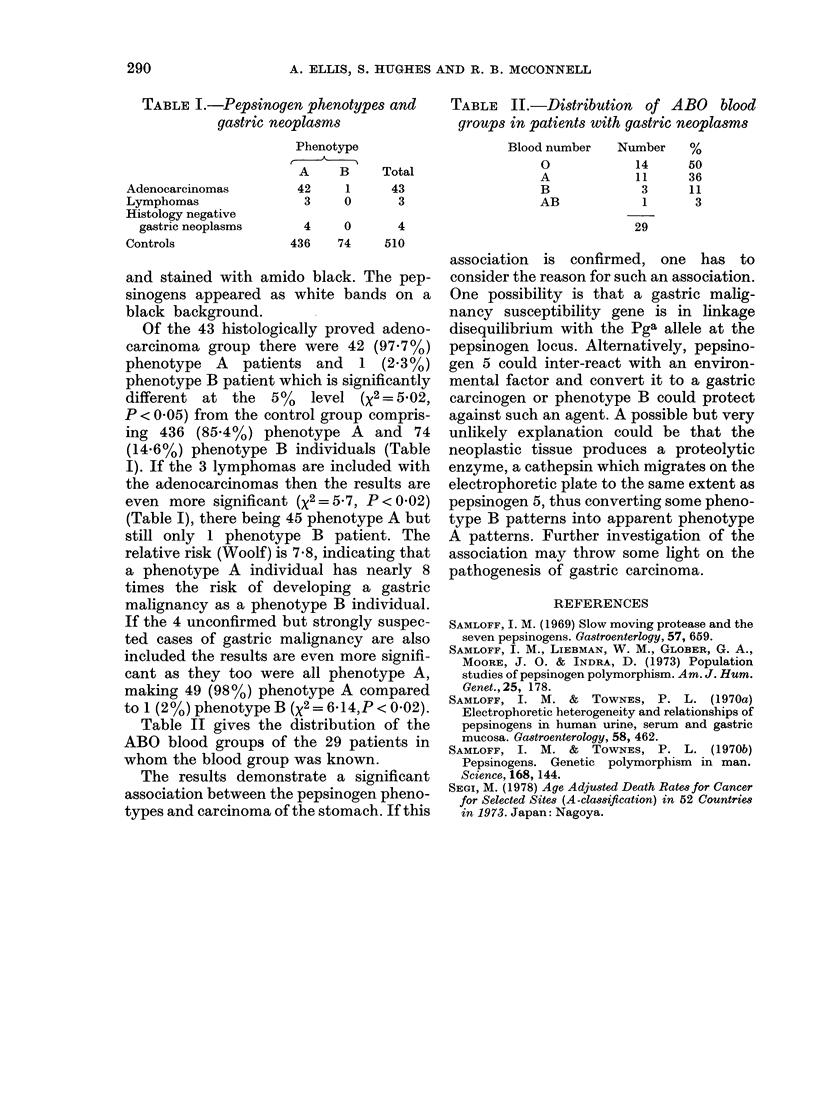# Gastric neoplasms and pepsinogen phenotypes.

**DOI:** 10.1038/bjc.1982.195

**Published:** 1982-08

**Authors:** A. Ellis, S. Hughes, R. B. McConnell


*Br. J. Cancer* (1982) **46**, 289

***Short Communication***

### GASTRIC NEOPLASMS AND PEPSINOGEN PHENOTYPES

A. ELLIS*, S. HUGHES†, AND R. B. McCONNELL‡

*From the *Department of Medicine, University of Liverpool, the †Gastroenterology Unit, Royal Liverpool Hospital and the ‡Gastroenterology Unit, Broadgreen Hospital, Liverpool*

Received 10 October 1981 Accepted 24 March 1982

A GENETIC POLYMORPHISM has been described of the human Group I pepsinogens (Samloff, 1969) characterized by the presence or absence of pepsinogen 5, called phenotypes A and B respectively (Samloff & Townes, 1970*a*). The inheritance of these phenotypes is controlled by 2 genes at an autosomal locus, phenotype B being inherited as an autosomal recessive trait (Samloff & Townes, 1970*b*). In an American Caucasian population, 84·4% were phenotype A and 15·5% were phenotype B (Samloff & Townes, 1970*a*). In contrast, 100% of a group of 229 Japanese individuals were phenotype A and no phenotype B persons were detected (Samloff *et al.*, 1973). Of interest is the fact that the Japanese also have the highest incidence of carcinoma of the stomach in the world (Segi, 1978). It was decided therefore to compare the distribution of pepsinogen phenotypes in a group of patients with gastric neoplasms and controls.

The patients were seen on the Gastroenterlogy Units and wards of the Royal Liverpool and Broadgreen Hospitals, Liverpool. Most were ascertained at endoscopy; the rest were seen after laparotomy for a positive radiological finding.

In 46/50 there was histological proof of a gastric malignancy. In 3 cases the radiologist and endoscopist were convinced of the malignant nature of the lesion but the histology of the endoscopic biopsy material was not confirmatory, and the patients refused operation. In one case an extensive gastric neoplasm with hepatic metastases was found at laparotomy and the patient's abdomen was closed without a biopsy being carried out. Of the 46 histologically proven neoplasms, 43 were adenocarcinomas of varying degrees of differentiation and 3 were lymphomas. The adenocarcinomas consisted of 24 men and 19 women. The age range of the men was 40–79 with a mean of 65 years, and women's ages ranged from 19 to 87 with a mean of 70 years. Most of the control urine samples were obtained from workers at Plessey Communications Ltd, Edge Lane, Liverpool, and the rest from nursing and university staff born in Merseyside.

A random sample of urine was obtained from each patient. After adding sodium azide, the urine was concentrated 20 times using Amicon B-15 miniconcentrators (Amicon Ltd, Woking, England). The concentrated samples were stored at 4°C until tested.

Pepsinogen phenotypes were determined by agar-gel electrophoresis, according to the method of Samloff (Samloff, 1969). Electrophoresis was carried out on glass plates 200 × 200 × 1 mm in 1·5% Difco Noble agar gel in 0·05M barbital buffer (pH 8·3) at 11 V/cm for $3\frac{1}{2}$ h in a TLC Mk II electrophoresis chamber (Shandon Southern Products Ltd, England) which was cooled by circulating iced water. The samples were mixed with an equal volume of warm 3% agar and pipetted into slots (1 × 10 mm) at the cathodal side of the plate. After the run the plate was immersed in acid-haemoglobin for 15 min, incubated in a humid atmosphere for 1 h at 37°C, fixed in acid-alcohol overnight

* Present address: Gastroenterology Unit, Broadgreen Hospital, Thomas Drive, Liverpool L14 3LB.
† Present address: Department of Medicine, Hope Hospital, Salford.

TABLE I.—*Pepsinogen phenotypes and gastric neoplasms*

| | Phenotype A | Phenotype B | Total |
|---|---|---|---|
| Adenocarcinomas | 42 | 1 | 43 |
| Lymphomas | 3 | 0 | 3 |
| Histology negative gastric neoplasms | 4 | 0 | 4 |
| Controls | 436 | 74 | 510 |

and stained with amido black. The pepsinogens appeared as white bands on a black background.

Of the 43 histologically proved adenocarcinoma group there were 42 (97·7%) phenotype A patients and 1 (2·3%) phenotype B patient which is significantly different at the 5% level ($\chi^2 = 5{\cdot}02$, $P < 0{\cdot}05$) from the control group comprising 436 (85·4%) phenotype A and 74 (14·6%) phenotype B individuals (Table I). If the 3 lymphomas are included with the adenocarcinomas then the results are even more significant ($\chi^2 = 5{\cdot}7$, $P < 0{\cdot}02$) (Table I), there being 45 phenotype A but still only 1 phenotype B patient. The relative risk (Woolf) is 7·8, indicating that a phenotype A individual has nearly 8 times the risk of developing a gastric malignancy as a phenotype B individual. If the 4 unconfirmed but strongly suspected cases of gastric malignancy are also included the results are even more significant as they too were all phenotype A, making 49 (98%) phenotype A compared to 1 (2%) phenotype B ($\chi^2 = 6{\cdot}14, P < 0{\cdot}02$).

Table II gives the distribution of the ABO blood groups of the 29 patients in whom the blood group was known.

TABLE II.—*Distribution of ABO blood groups in patients with gastric neoplasms*

| Blood number | Number | % |
|---|---|---|
| O | 14 | 50 |
| A | 11 | 36 |
| B | 3 | 11 |
| AB | 1 | 3 |
| | 29 | |

The results demonstrate a significant association between the pepsinogen phenotypes and carcinoma of the stomach. If this association is confirmed, one has to consider the reason for such an association. One possibility is that a gastric malignancy susceptibility gene is in linkage disequilibrium with the $Pg^a$ allele at the pepsinogen locus. Alternatively, pepsinogen 5 could inter-react with an environmental factor and convert it to a gastric carcinogen or phenotype B could protect against such an agent. A possible but very unlikely explanation could be that the neoplastic tissue produces a proteolytic enzyme, a cathepsin which migrates on the electrophoretic plate to the same extent as pepsinogen 5, thus converting some phenotype B patterns into apparent phenotype A patterns. Further investigation of the association may throw some light on the pathogenesis of gastric carcinoma.

### REFERENCES

SAMLOFF, I. M. (1969) Slow moving protease and the seven pepsinogens. *Gastroenterlogy*, **57**, 659.

SAMLOFF, I. M., LIEBMAN, W. M., GLOBER, G. A., MOORE, J. O. & INDRA, D. (1973) Population studies of pepsinogen polymorphism. *Am. J. Hum. Genet.*, **25**, 178.

SAMLOFF, I. M. & TOWNES, P. L. (1970*a*) Electrophoretic heterogeneity and relationships of pepsinogens in human urine, serum and gastric mucosa. *Gastroenterology*, **58**, 462.

SAMLOFF, I. M. & TOWNES, P. L. (1970*b*) Pepsinogens. Genetic polymorphism in man. *Science*, **168**, 144.

SEGI, M. (1978) *Age Adjusted Death Rates for Cancer for Selected Sites (A-classification) in 52 Countries in 1973*. Japan: Nagoya.